# COVID-19 and race: Protecting data or saving lives?

**DOI:** 10.1177/1470785320946589

**Published:** 2020-09

**Authors:** Richard Webber

**Affiliations:** OriginsInfo Ltd, UK; University of Newcastle, UK

**Keywords:** COVID-19, ethnicity, inference, names, origins

## Abstract

This article uses the COVID-19 pandemic to demonstrate how our understanding of ethnic inequalities could be improved by greater use of algorithms that infer ethnic heritage from people’s names. It starts from two inter-connected propositions: the effectiveness of many public sector programs is hampered by inadequate information on how differently different ethnic groups behave, and anxiety over how to discuss matters to do with race inhibits proper evaluation of methodologies which would address this problem. This article highlights four mindsets which could benefit from challenge: the officially sanctioned categories by which ethnic data are tabulated are too crude to capture the subtler differences which are required for effective communications; while self-identification should continue to drive one-to-one communications, it should not preclude the use of more appropriate methods of recording ethnic heritage when analyzing data for population groups; public servants often fail to recognize the limitations of directional measures such as the Index of Multiple Deprivation as against “natural” classifications such as Mosaic and Acorn; and in their quest for predictive accuracy statisticians often overlook the benefit of the variables they use being “actionable,” defining population groups that are easy to reach whether geographically or using one-to-one communications.

## Why it is so difficult to talk about race?

There are two particular issues which we believe merit the attention of the market research sector. The first is understanding why it should be so difficult to engage in public discussion of the behaviors that characterize different ethnic groups. The second relates to evidence. In our experience, few public servants are as well supported as they would like to be with evidence of how different minority populations use the services they are responsible for delivering. Why should this be?

It frustrates us that these two problems are so inter-connected. We cannot talk intelligently about race because we cannot access the information we need. We cannot access the information we need because it is so difficult to talk about race.

This circularity raises fundamental questions, currently inadequately debated, about how we collect, structure and maintain data revealing people’s ethnic backgrounds. Data protection protocols do not help and they should be included in any debate on this topic as reducing restrictions on access to data would undoubtedly save many lives.

Over the last 6 months, the salience of this issue has increased significantly. The COVID-19 crisis has brought into much sharper focus the proposition that better access to data would enable us to tackle ethnic inequalities more effectively.

It is not that the press has not covered the uneven impact of COVID-19, or race more generally. For example, on 8 June 2020, much of the front page of *The Times* was given over to the toppling of a statue of Edward Colston in Bristol; Page 2 featured criticism by Black leaders of a report published by Public Health England on the link between COVID-19 and ethnicity; and the back page led with the obstacles Black soccer players face when applying for management positions.

But there is much less discussion of the likely reasons for these differences, other than politically “safe” explanations, such as poorer health and more household overcrowding about which little can be done, at least not in the short term. Nor is there significant coverage of the more nuanced messages that local authorities might use to communicate with different minority groups. It seems everyone is frightened of saying something that will cause offense. It is safer to keep quiet.

The obstacles that prevent public servants and executives in commercial organizations from obtaining evidence to better understand differences in ethnicity can be summarized under four distinct headings each of which it may be time for the market research industry to start to challenge:

- The industry analyses ethnicity using too few categories.
- We focus too much on self-identification of ethnicity.
- We rely too much on directional rather than natural classifications.
- We overlook the need for actionability.

## Problems associated with the use of too few categories

**Figure 1.**
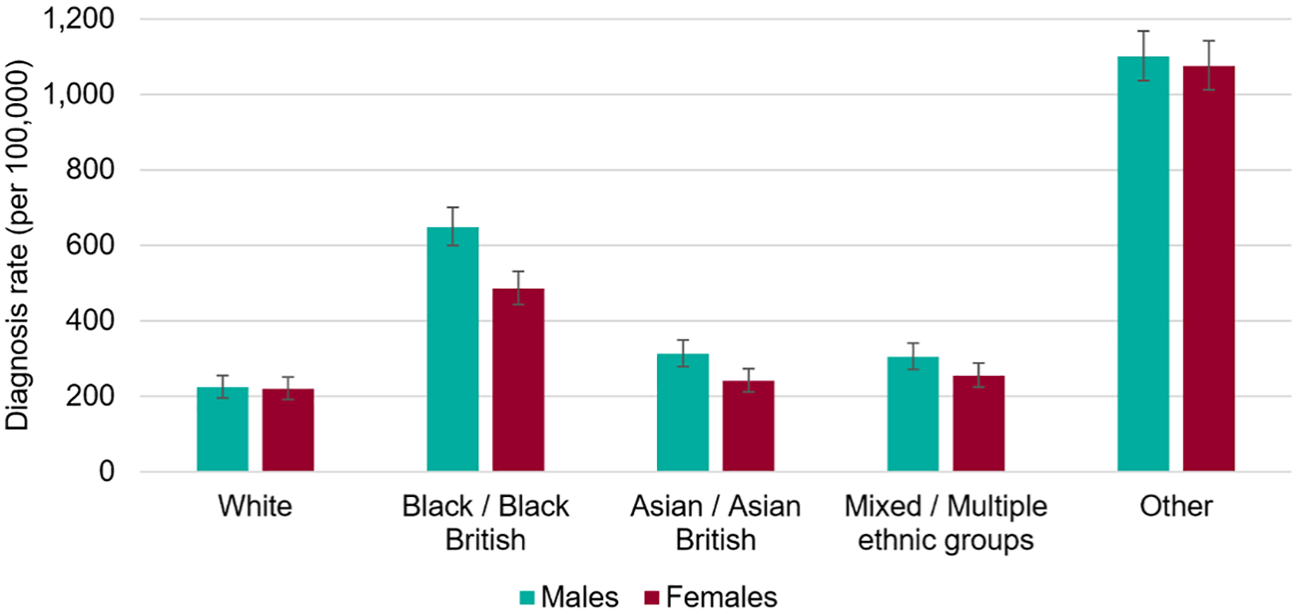
Age standardized COVID-19 diagnosis rates by ethnicity and sex, as of 13 May 2020. Source: Public Health England Second Generation Surveillance System.

Figure 1 is taken from what was billed as a definitive report into COVID-19 and ethnicity published by Public Health England (PHE) in June 2020.

It compares the morbidity rates of the white population and those of four other ethnic groups. Revealing though the table is, it is insufficient to tease out many possible reasons for unequal levels of morbidity, largely because each category combines ethnic groups whose circumstances and behaviors are very different.

Using just a subset of sub-groups within the Origins classification, Table 1 identifies variations between the ethnicity of staff, patients, and residents living within the natural catchment area of the Royal Free Hospital, London. It reveals that while the ratio of Tamil staff to Tamil patients is three times the hospital average, the corresponding ratio for Bangladeshis and Somalis is just one third of the hospital average. Groups that to Western Europeans may seem very similar have very different occupational preferences.

**Table 1.** Percentage distribution of staff at the Royal Free Hospital by various categories of ethnic group, 2010.

Origin	% staff	% patients	% residents	Ratio of staff to patients	Ratio of staff to residents
Black Caribbean	0.57	0.16	0.21	3.56	2.71
Tamil	1.40	0.48	0.69	2.92	2.03
Sikh	0.77	0.34	0.38	2.26	2.03
Irish	6.45	6.81	6.39	0.95	1.01
Pakistani	1.74	4.61	3.88	0.38	0.45
Turkish	0.77	2.21	2.67	0.35	0.29
Bangladeshi	0.72	2.20	1.69	0.33	0.43
Somali	0.23	0.85	0.58	0.27	0.40

One complaint reported in the edition of *The Times* on 8 June is that the PHE report ignores the contribution that occupation makes to different levels of vulnerability to COVID-19. It is generally known that Filipinos are far more likely to work in care services than people of Chinese heritage; Nigerians and Ghanaians are more likely to work in care homes than North Africans; Tamils are more likely to work as supermarkets cashiers than Pakistani Muslims; and mini-cab drivers and people who deliver take-aways are disproportionately drawn from Britain’s Muslim community.

The impact of different rates of morbidity experienced by people in these occupations is lost when data are aggregated to just half a dozen categories. The most striking evidence from Figure 1 is the disproportionate vulnerability to COVID-19 of the ethnic groups “Other,” a grouping which, according to the PHE report, has a 10 times higher propensity to die from the virus than white Britons.

For a condition where genetics or diet is thought to be a potential contributor to uneven morbidity, it may not be helpful to group together as “White Other” people of such different origins as Colombians, Romanians, Finns, Serbs, and Turks, notwithstanding the similar colors of their skins.

The problem of insufficient categories is equally evident in Table 2 which shows the origins of hospital patients in Kent in 2019.

**Table 2.** Ethnicity codes of Kent hospital patients, 2019.

All Kent patients, 2019	(,000 patients)	%
Total	1,664	100.0
White British or Irish	1,426	85.7
Declined or not stated	118	7.1
Not white British or Irish	108	6.5
Six defined BAME groups	16	1.0
Other white	58	3.5
Any other non white	27	1.6
Mixed heritage	7	0.4

BAME: Black, Asian and minority ethnic.

Of the 6.5% of patients who are recorded as not white British or Irish more than half, 3.5%, are recorded as “Other white,” and a further 1.6% of patients have no classification other than they are not white. In Kent, therefore, the ethnic classification field held in databases of episode statistics fails to deliver a clear ethnic origin for over three quarters of non-white British patients.

What then are the origins of the mind-set that it is acceptable to divide Britain’s Black, Asian and minority ethnic (BAME) community into just five or six categories? I would contend that it originated, not unreasonably, from the limitation imposed by traditional fieldwork practices with their restricted sample sizes in an era when there were many fewer immigrant groups. A taxonomy appropriate for that purpose at that time has not evolved in response to the opportunities big data provide to analyze far larger numbers of records, not least in the field of public health. Whereas a 40,000 respondent fieldwork survey is unlikely to generate enough Albanians or Filipinos to create a statistically significant sample, the 15,000,000 record Health Episode Statistics database has more than enough of both.

Many people may wonder how it is possible to code such a large number of records, in detail and with an adequate degree of accuracy. Yet, it is now over 20 years ago that the health service first used name recognition algorithms such as Sangra and Nam Pehchan, devised by the School of Oriental and African Studies and by Bradford City Council respectively, specifically to attribute an inferred ethnicity to health service patients with South Asian names.

If, as I still hope will happen, the PHE’s surveillance data can be enriched with Origins, a name recognition algorithm which operates across all ethnic groups and not just South Asians, PHE can then compare morbidity data across a set of 50 or so recognizably distinct groups based on the ethno-cultural background of the patient’s forebears.

## The undue assumption that ethnicity can only be established through self-identification

It is difficult to consider the mind-set that results in the use of too few categories without considering a separate set of problems relating to the entitlement we give to data subjects regarding their right to specify their ethnicity. These problems originate from a failure to distinguish two very different ways in which subject data are used.

Most data subjects reveal information about themselves on the expectation that it will be used to determine the manner in which they are treated. It is this expectation that underpins a person’s right to define their own ethnic identity that is enshrined in data protection legislation and hence dominates the way the research industry thinks about data on ethnicity. It also governs the industry’s perceptions regarding accuracy, as though, if we were able to drill down far enough into a person’s mind or body, we would be able to locate evidence of a single correct classification, one that could capture their ethnicity as unambiguously as their date of birth can record their age. We assume that it is only the data subject that knows what the accurate answer to this question is.

No one would dispute the legitimacy of this entitlement in situations where data are used to drive the manner in which the data subject are addressed or treated, for example, in a doctor’s surgery, in a hospital, by an employer, and at a school or when using social media.

But it is not axiomatic that such entitlement should extend to the use of personal data which are collected exclusively for statistical analysis. When epidemiologists use aggregate clinical data to discover the link between ethnicity and morbidity, why should we require them to restrict their analyses to cases where the data subject have supplied such data, or that the self-identification given by the data subject should be allowed to override other objective methods of deriving ethno-cultural origin?

The Black Minnesota resident, George Floyd, did not get murdered by a police officer because he self-identified as Black. He was murdered because he was perceived as Black by the police officer who killed him. Protesters claimed on Page 2 of the 8 June 2020 edition of *The Times* that BAME hospital staff were more vulnerable to COVID-19 infection because of the racism they are subjected to from their colleagues. The implications of both examples are clear. If you are from a BAME group, other people’s perceptions of your identity have as powerful an effect on your life experience as your perception of your own identity.

The market research industry should continue to collect and retain data based on self-identification but that does not mean that it should restrict the ethnicity data it holds to the data subject’s self-identification. Wherever possible, self-identified ethnicity should be recorded in a separate data field on a database from the identity inferred using a name recognition algorithm.

## Over-reliance on directional rather than natural classifications

The third constraint on the ability of research to generate outputs relevant to ethnicity relates to the over-reliance on directional at the expense of natural classifications.

Over 40 years ago, when I was involved in the creation of first Acorn and then Mosaic, we did something which at that time was more revolutionary than we realized, and which is still not as common as it might be, even today. We gave data the right to speak for itself.

What this means is that we relied on a computer algorithm, using a very early form of artificial intelligence, to decide for itself what should be the various categories of postcode that should be used for the analysis of consumer behavior.

**Figure 2.**
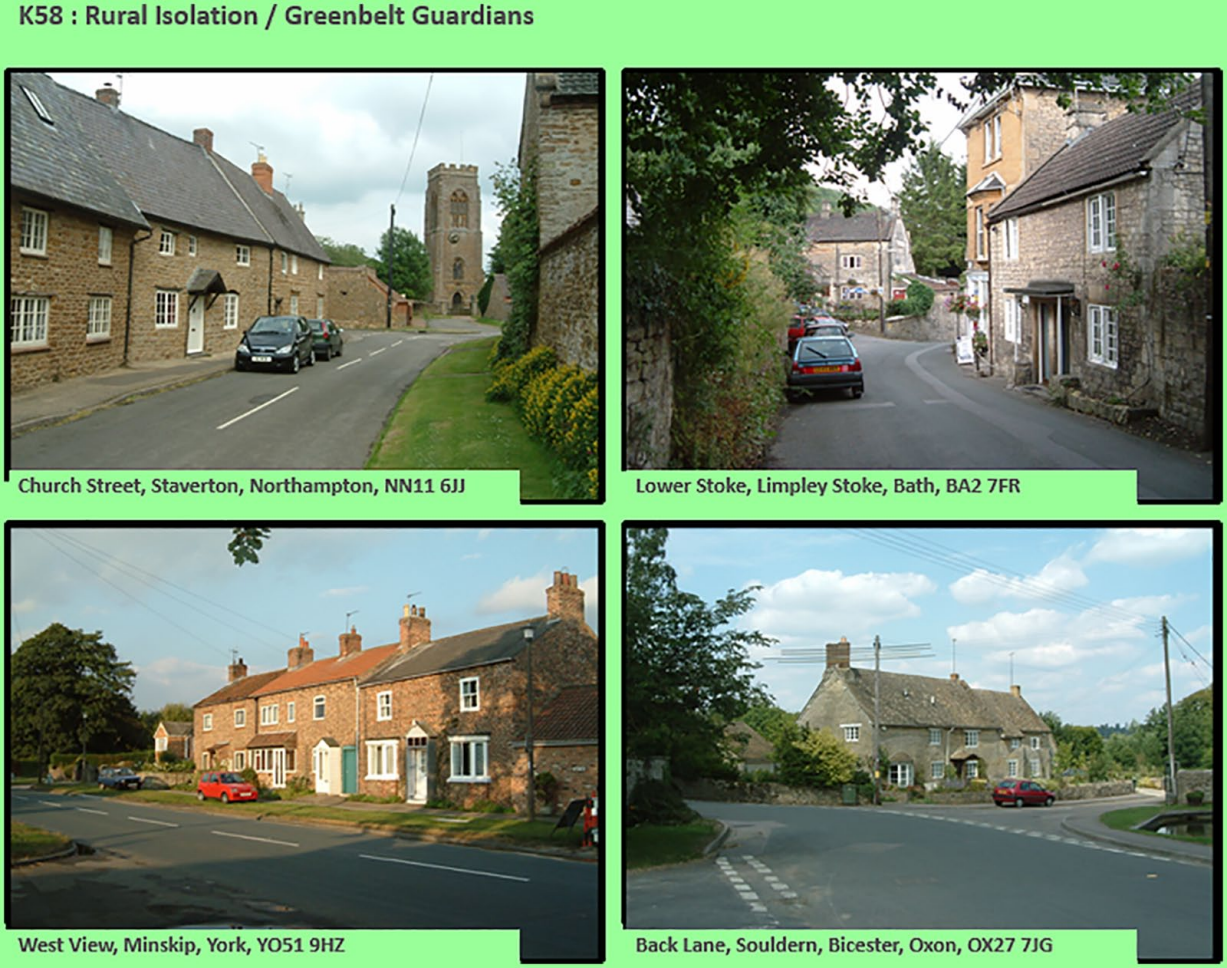
Mosaic type K58, “Greenbelt Guardians.”

If the computer were to find a group of neighborhoods broadly similar across a wide range of different statistical measures, who were we to overrule it? If it found a set of neighborhoods which corresponded to the description “*Greenbelt Guardians*” then so be it, “*Greenbelt Guardians*” should become a category (Figure 2). As nurse maidens our job was merely to come up with suitable names, photographs, and descriptions for each category.

By contrast with this “natural” approach, which also happens to be the basis for any successful search engine, the instinctive response of public servants is to employ a “directional” taxonomy. For example, when citizens were first classified by social class, it was by means of their occupations which had been placed into categories according to their average life expectancy. When government decided to classify neighborhoods, it decided that it should place them into deciles according to their score on an overall measure aligned with deprivation.

Social class and the Index of Multiple Deprivation are both directional or purposive constructs, that is to say they were designed with the objective of addressing specific issues, namely unequal life expectancy and unequal social and economic opportunities. Both have proved their usefulness for these particular purposes but neither deliver as powerful discrimination on other measures of behavior as do natural classifications, both because they are one-dimensional and because they can support fewer categories.

The news item on Page 2 of the 8 June issue of *The Times* is one of many instances where deprivation is cited as a potential contributor to the high death rates from COVID-19 among BAME citizens. Table 3, which cross tabulates two natural classifications, Mosaic and Origins, reveals the very different types of deprived area in which different vulnerable minorities live. The minorities most likely to live in the Mosaic group “Lowest status inner city social housing” include Eritreans, Vietnamese and Turks, ethnic groups that do not fit readily into the categories used in the PHE study. Pakistani Muslims and Bangladeshis, by contrast, are much more likely to live in the Mosaic classification “Lowest status older terraces.”

**Table 3.** Cross tabulation between selected Mosaic and Origins types.

	Lowest status inner city social housing	Lowest status older terraces	Combined	Index (combined)
**UK adults**	**4.4**	**0.9**	**5.3**	**100**
Bangladeshi	16.9	30.2	47.1	889
Pakistani Muslim	30.5	7.2	37.7	711
Eritrean	36.0	0.8	36.8	694
Vietnamese	28.4	3.3	31.7	598
Ghanaian	30.5	0.8	31.3	591
Turkish	19.0	1.6	20.6	389
Black Caribbean	16.0	2.2	18.2	343
Hindu Indian	3.5	7.2	10.7	201
Sikh	3.1	6.9	10.0	189

Bold values indicates UK totals.

Areas with the most unfavorable scores on the Index of Multiple Deprivation can include very different types of neighborhood which attract very different ethnic groups. Thus in the event that central government were to act to address disparities in housing conditions, it would need to develop very different policies in neighborhoods with large Pakistani and Bangladeshi populations than would be appropriate in area with large numbers of Turks and Vietnamese. Different policies would be needed for run down streets built in the 19th century in Oldham and Bradford than in post 1945 flats owned by councils such as Lambeth and Tower Hamlets.

**Figure 3.**
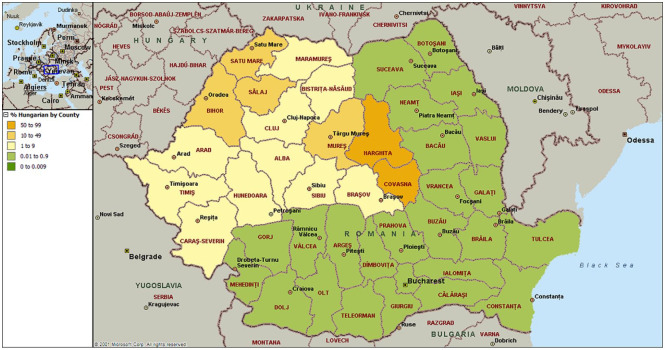
Where ethnic Hungarians live in Romania.

Figure 3 shows the percentage of Romanian telephone subscribers in 2009 who bear names suggesting Hungarian ethno-cultural heritage. The pattern reflects decisions made after the collapse of the Austro-Hungarian empire and is relevant to London Councils such as Brent and Harrow which are home to the largest numbers of UK migrants from Romania. It is not just in Africa that current national boundaries misalign with those based on language or culture.

Quite apart from offering more nuanced categories, a name-based classification allows more flexible grouping of the categories that it generates. Thus, at a coarser level, instead of grouping North Africans with Black sub-Saharan Africans, they naturally group themselves with Arabs, Turks, and Iranians. This is because the names they share, unsurprisingly, are markers of a common faith. In Britain, the fact that they worship in the same mosques is highly relevant to the use of any results of analysis of Covid-19 by ethnicity for the design of communications strategies for reaching high risk minority groups.

One of the common problems analysts have with directional classifications is the difficulty calculating incidence rates. For example, when PHE had collected information on the number and percentage of cases of COVID-19 among the group “Others” it needed to compare this with corresponding size of the group “Others” in the base population. As can be seen from the following statement the PHE report on ethnicity and COVID-19 recognised this constituted a problem in accurate estimation of incidence rates by ethnic group:

The rates in the Other ethnic group are likely to be an overestimate due to the difference in the method of allocating ethnicity codes to the cases data and the population data used to calculate the rate.

## Our tendency to overlook the need for actionability

In the early days of Acorn, marketeers told me that they liked its “actionability.” That was not a word I had heard before. What I later understood it to mean was that if, for example, the National Trust was to find that *Greenbelt Guardians* was the segment it performed best in, the Trust could then identify the postcodes, the door-to-door delivery areas, and the high streets where *Greenbelt Guardians* were to be found. The Trust could use the same categories for targeting communications and for analysis.

This is why in terms of actionability it makes sense to associate the category North Africans with other Muslim groups. They go to the same mosques. Separating Sikhs from Hindu Indians also makes sense—they worship at different temples.

When the health communications organization Dr Foster was charged with improving diabetes screening among Slough’s South Asian community, it initially set up information centers in supermarket car parks and posters in doctors’ surgeries. In time, Dr Foster discovered that neither channel was as effective as asking elders in South Asian temples and mosques to pass on their message to their worshippers. It made the elders feel good to be seen as representatives of a government organization.

It seems inconceivable that a COVID-19 communication strategy that targets high-risk groups would not benefit from the involvement of religious leaders. This is one reason why it is useful for categories to be strongly aligned with religion.

In other words, categories such as Tamil, Sikh, Hindu Indian and Pakistani Muslim tend to be more actionable than ones based on nationality, such as Sri Lankan, Indian, Bangladeshi and Pakistani. After all, British Sikhs do not divide themselves according to which side of the partition line their forebears lived in the Indian sub-continent prior to 1947.

But to save lives local authority public health departments need to be served with tools to target their communications more efficiently.

**Figure 4.**
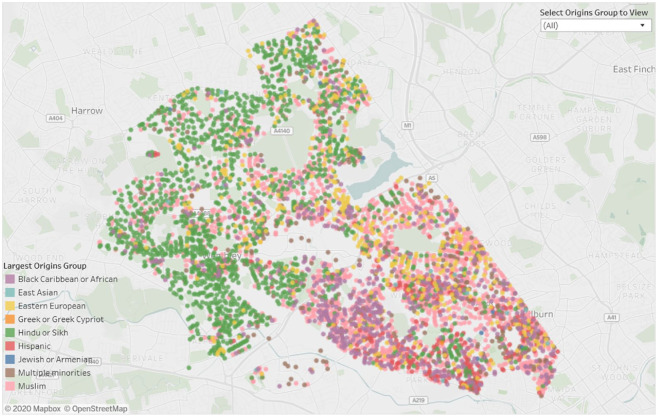
Distribution of ethnic communities in Brent.

Figure 4 shows postcode data for Brent for March 2020, individual postcodes being colored according to their most numerous minority—the green postcodes in the north are mostly Hindu or Sikh; the pink dots in the east are Muslim; and the purple dots in the south are Black Caribbean communities. Note that using a natural classification, it is possible to map ethnicity using a finer level of categorisation than using a directional classification; the units of geography are finer and the data can be updated on as frequent a basis as is necessary.

One other interesting property of a classification of neighborhoods based on the largest minority is that it may help public servants target citizens that are otherwise most hard to reach using conventional techniques. Although we cannot know for sure, it is not an unreasonable assumption that BAME populations most at risk of COVID-19 are those who live in close proximity to other members of their own community.

Like many previous health studies, the PHE report structures data in the hope of finding explanations for the unequal incidence of the virus, whether in terms of genetics, health or deprivation, perhaps without any conscious recognition of the practical benefits of including a categorisation which would support targeted communications to high-risk groups.

## Ethnicity, GDPR, and the Information Commissioner’s Office

The last 5 years has seen an increase in the number of statutory obligations imposed on large businesses to demonstrate fairness both in respect of employee recruitment and in customer treatment. During this period, there has been a very sharp drop in the use of name recognition systems for either purpose. This decrease can be attributed in part to the burdensomeness of obligations to make a case for public interest exemption, which most non-government organizations feel unable to do, to comply with restrictions surrounding the use of “sensitive personal data” and to prepare a data protection impact assessment for each project.

Most organizations are deterred from commissioning this type of work on the grounds of legal uncertainty and potential reputational risk. The easiest option is to do nothing and, if challenged, to justify inaction on grounds of requirement to comply with their obligations under General Data Protection Regulation (GDPR). For those organizations brave enough to proceed in the face of these regulatory headwinds, our estimate of the resource cost of undertaking the analysis is typically in the region of one tenth the resource involved in obtaining internal regulatory clearance whether in relation to making the argument for public interest exemption, preparing a Data Protection Impact Assessments or setting out data processing protocols in relation to Sensitive Personal Data.

In terms of elapsed time, our organization’s rule of thumb is that acquiring approvals of this sort take 20 times as much time as it takes to do the analysis.

In addition to this additional resource cost, there is the anxiety regarding potential reputational risk in an environment which is so protective of personal data and the possibility of blame being attached not just to the organization but to the individual who sanctioned the analysis.

Such concerns and costs would be understandable if the data were to be used for personal communications or if the data had not already been created in an anonymised or aggregated form which made it impossible to identify the data subject. But in almost every instance such concerns are not the case and there is no material risk of any individual or their ethnicity being identified.

In normal times, the stated practice of the Information Commissioner’s Office (ICO), which is to follow public opinion on this matter, would appear reasonable. Yet in 2020, we are not in normal times. Although COVID-19 has been responsible for the death of over 40,000 British citizens, we are aware of no statement from the ICO adapting the provisions of GDPR legislation to take this abnormal situation into account.

Likewise, while we are unaware of any public demonstrations to protect personal data against abuse, we have on the weekend prior to the day of the 8 June lecture witnessed widespread demonstrations under the umbrella of the Black Lives Matter movement.

Although the initial spark for these demonstrations was the death of George Floyd, the failure of the PHE report to satisfy the expectations of BAME leadership has contributed to a widespread belief that not enough is being done by government to combat the unequal opportunities under which the BAME population labors.

Whether opportunities become more equal will depend on the effectiveness of the various statutory measures the government has already put in place to require organizations to demonstrate fairness, to report existing inequalities, and to produce statistical evidence of the effectiveness of measures taken to reduce inequalities. Sadly, it is our view at the present time that the ICO shows no awareness of the role of data in satisfying the demands of the Black Lives Matter campaign or the government’s own policies in respect of inequalities. Nor does the ICO appear to show awareness of how it might satisfy these objectives.

Two very simple changes would do a lot to further these aims. The first is that when used in support of an organization’s statutory obligations in relation to equality, the use of name recognition algorithms should be deemed as satisfying a public interest requirement.

The second is that the codes generated by name recognition systems should no longer be deemed to constitute protected personal data in situations where these data were accessible only in the form of aggregated statistics or in a de-personalized and anonymous form from which it would be impossible to reverse engineer the identity or ethnicity of individual data subjects.

